# The Influence of Bilingual Language Exposure on the Narrative, Social and Pragmatic Abilities of School-Aged Children on the Autism Spectrum

**DOI:** 10.1007/s10803-022-05678-w

**Published:** 2022-10-12

**Authors:** Myriam L. H. Beauchamp, Stefano Rezzonico, Terry Bennett, Eric Duku, Stelios Georgiades, Connor Kerns, Pat Mirenda, Annie Richard, Isabel M. Smith, Peter Szatmari, Tracy Vaillancourt, Charlotte Waddell, Anat Zaidman-Zait, Lonnie Zwaigenbaum, Mayada Elsabbagh

**Affiliations:** 1 McGill University, Montréal, Canada; 2 Université de Montreal, Montréal, Canada; 3 McMaster University, Hamilton, Canada; 4 University of British Columbia, Vancouver, Canada; 5 Dalhousie University and IWK Health, Halifax, NS Canada; 6 Centre for Addiction and Mental Health, University of Toronto, The Hospital for Sick Children, Toronto, Canada; 7 University of Ottawa, Ottawa, Canada; 8 Simon Fraser University, Burnaby, Canada; 9 Tel Aviv University, Tel Aviv, Israel; 10 University of Alberta, Edmonton, Canada

**Keywords:** Autism spectrum, Bilingualism, Narratives, Social skills, Pragmatic language

## Abstract

We examined the narrative abilities of bilingual and monolingual children on the autism spectrum (AS), whether bilinguals presented stronger social and pragmatic language abilities compared to monolinguals, and the link between narrative, social, and pragmatic language abilities.

The narrative, social, and pragmatic language skills of school-aged bilinguals (n = 54) and monolinguals (n = 80) on the AS were assessed using normed measures. Language exposure was estimated through a parent questionnaire.

Bilinguals performed similarly to monolinguals on measures of narrative, social, and pragmatic language skills. However, balanced bilinguals performed better on a nonliteral language task.

Overall, results indicate that bilingual children on the AS can become as proficient in using language as monolinguals and may enjoy a bilingual advantage.

## Background

Bilingualism is the ability to use more than one language in everyday life regardless of one’s level of proficiency or of the modalities (i.e., reading, speaking, or listening) in which either language is used (Grosjean, 1993). Globally, bilingualism is the rule rather than the exception (Grosjean, 1982). However, within the context of language and developmental research, monolingualism has generally been viewed as the norm. Nevertheless, research has found that overall, neurotypically developing (ND) bilingual children follow similar developmental trajectories to those of their monolingual peers and are often able to keep pace with those peers, at least in their stronger language (i.e., the language in which they are most proficient), and sometimes in both of their languages (Beauchamp & McLeod, 2022; MacLeod et al., 2013; Thordardottir, 2011; Thordardottir, 2015, also see Paradis et al., 2021, for review). However, differences, often subtle, can exist between these two groups. For example, bilingual children may have smaller vocabularies in one language, although when both languages are considered, these differences often disappear (Pearson et al., 1997). Morphological markers, such as gender markers in French, can also be more challenging for some bilingual children to acquire fully (Eichler et al., 2012). Conversely, ND bilingual children are able to acquire certain aspects of language more quickly than their monolingual peers, especially when able to use knowledge from one language to support acquisition of another (Yip & Matthews, 2000).

Differences between bilinguals and monolinguals regarding language acquisition should not be viewed as deficits but rather as variations from the monolingual norm that are generally influenced by a bilingual speaker’s language experiences. Factors such as the amount of exposure children receive to each of their languages (Thordardottir, 2011, 2015, 2019), the language(s) used at school and at home (Gathercole & Thomas, 2009), and whether a language is a majority or minority language (MacLeod et al., 2013) influence speakers’ proficiency in each of their languages.

One area of particular interest when investigating the similarities and differences between bilinguals and monolinguals is narrative skills; that is, the ability to talk about a series of related events that occur in a chronological order, which generally include a goal and actions to reach that goal (Baixauli et al., 2016; Heilmann et al., 2010). Such skills are an important aspect of language ability and serve a vital communicative function (Norbury et al., 2013). Strong narrative skills may also foster psychological and emotional identity (Bruner, 2003), and in preschool-aged children they are positively linked to academic achievement (O’Neill et al., 2004).

Narratives can be divided into two broad components: microstructure and macrostructure. Microstructure refers to the structural details of language, such as the number of words, vocabulary diversity, sentence structure and complexity, and mean length of utterance (MLU). Macrostructure includes broader aspects of the narrative such as its coherence or overall structure (i.e., does the story have a clear beginning, middle, and end?; are there characters and are they well developed?; is there a goal?) as well as the appropriate production of temporal and causal information, and the cohesion of the narrative (i.e., does the story use appropriate referencing?, does the story follow a logical order?; Baixauli et al., 2016; Heilmann et al., 2010). Proficient storytelling places high demands on both linguistic knowledge (i.e., lexical and grammatical knowledge) and cognitive abilities (Losh & Capps, 2003). It also necessitates strong discourse, social and pragmatic skills (i.e., the way in which context influences meaning, including in social context; Ketelaars et al., 2016) because the context within which a story is produced (i.e., listener characteristics and the goal of the story) influences the organization of the story as well as the wording. Consequently, being a skillful storyteller requires the ability to infer, take others’ perspectives, and think holistically.

Difficulties in these domains may explain some of the challenges that individuals on the autism spectrum have with narratives. Indeed, these individuals often struggle with narrative production, with macrostructure generally being more impaired than microstructure (Baixauli et al., 2016; Geelhand et al., 2020). In their meta-analysis, Baixauli et al., (2016) found weaker narrative performances in children and adolescents on the autism spectrum compared to ND individuals, especially when examining measures of coherence. Poorer performances have also been reported in individuals on the autism spectrum across many microstructure measures (numbers of words and utterances, different words, MLU, and syntactic complexity) compared to ND individuals, although this domain of narrative is more heterogeneous than is macrostructure. It is notable that, in children on the autism spectrum, difficulties with microstructure are not necessarily secondary to a language disorder. Indeed, children on the autism spectrum without a language disorder have been found to show similar microstructure abilities to those of their peers with a developmental language disorder, and significantly lower microstructure scores than their ND peers (Norbury et al., 2013). Thus, abilities beyond formal language knowledge appear to play an important role in microstructure skills for individuals on the autism spectrum when producing a narrative.

Deficits in social and pragmatic language skills may, in part, explain the narrative difficulties of individuals on the autism spectrum. Specifically, deficits in Theory of Mind (i.e., the ability to understand our own minds and those of others; Carlson et al., 2013) and weak central coherence (i.e., focusing on details rather than the “big picture”; Frith & Happé 1994), both of which are linked to social and pragmatic abilities, have been argued to underpin challenges with narrative skills (Baixauli et al., 2016; Siller et al., 2014). In line with these findings, using cross-sectional data from the Pathways study, we also found previously that in 8-year-old children on the autism spectrum, narrative abilities were not only associated with nonverbal cognitive and language skills, but also with social skills (Volden et al., 2017). Thus, weaker social and pragmatic language skills appear to be linked to weaker narrative abilities. For example, difficulties with perspective taking could influence one’s ability to tell a story from different characters’ perspectives and make referencing more challenging. Difficulties in central coherence could also influence one’s ability to weigh the importance of the different events and characters appropriately within a story. Consequently, when children on the autism spectrum produce a narrative, they tend to include information that is irrelevant, making their stories longer and less clear than those of their ND peers (Norbury et al., 2013), a finding similar to that reported in children with a pragmatic language impairment (Ketelaars et al., 2016).

A growing body of literature examining ND bilingual children’s narrative abilities suggests that microstructure is more sensitive to the amount of exposure that a child has received to the language of testing, whereas macrostructure skills tend to be preserved, even when children are tested in their weaker language (Hipfner-Boucher et al., 2015). However, macrostructure is influenced by children’s underlying language abilities, and bilingual children do require some competence (i.e., microstructure skills) in the language of testing to produce a strong macrostructure (Rezzonico et al., 2016).

There is limited research into bilingual language development in children on the autism spectrum. This reality may reflect the prevalent misconception that bilingualism is an additional burden and increases language deficits in these children (Kay-Raining Bird et al., 2012; Kremer-Sadlik, 2005; Yu, 2013). However, in line with studies examining the language abilities of ND bilinguals, children on the autism spectrum from various age groups and with varying language and cognitive abilities can reach similar levels of proficiency to those of their monolingual peers (Beauchamp et al., 2020; Hambly & Fombonne, 2012; Kwok et al., 2015; Ohashi et al., 2012; Peterson et al., 2012). Evidence is also emerging that bilingual children on the autism spectrum, particularly those of preschool age, may have stronger social abilities than their monolingual peers (Hambly & Fombonne, 2012; Valicenti-McDermott, et al., 2015).

There is also little research regarding the ﻿examining narrative abilities of bilingual children on the autism spectrum. In a recent study, the narratives of bilingual school-aged children on the autism spectrum compared to those of their monolingual peers on the autism spectrum, and to their ND bilingual and monolingual peers (N = 20, n = 5 per group), were examined (Hoang et al., 2018). Findings showed no significant group differences on measures of microstructure. However, there were marginal group differences on measures of macrostructure, with a large effect size when comparing monolingual children on the autism spectrum to their monolingual ND peers, and a small effect size when comparing bilingual children on the autism spectrum to their bilingual ND peers, with ND children presenting stronger abilities than their peers on the autism spectrum in both instances.

Although these findings are interesting, more research is required to better understand the narrative abilities of bilingual children on the autism spectrum. This line of research is particularly important given the link between narrative skills and both social and pragmatic skills on the one hand, and between bilingualism and social and theory of mind abilities on the other hand. Indeed, previous studies have found that being a bilingual speaker may lead to stronger abilities in the area of theory of mind in neurotypically developing children (Farhadian et al., 2010; Goetz, 2003), and to stronger social skills in children on the autism spectrum (Hambly & Fombonne, 2012). Given the link between social and narrative skills, it is possible that bilingualism may lead to better narrative abilities in children on the autism spectrum.

Our aims for the current study were to increase our understanding of similarities and differences in the narratives produced by bilingual and monolingual children on the autism spectrum, and of the links between narrative abilities and both social and pragmatic skills.

Based on the link between social skills and narratives on the one hand (Volden et al., 2017), and between bilingualism and social skills on the other (Hambly & Fombonne, 2012), we predicted that children in the bilingually exposed group would have stronger performances than their monolingually exposed peers on measures of macrostructure. However, given some evidence from ND literature (Gollan et al., 2007; Paradis et al., 2008), we also expected that bilingually exposed children might obtain slightly lower scores on measures of microstructure. Additionally, we predicted that bilingual children would perform better on measures of social and pragmatic language. Finally, we predicted that scores on measures of social and pragmatic language skills would be linked to stronger narrative skills, thus replicating some of the earlier results with this cohort when they were 8 years old (Volden et al., 2017), particularly for macrostructure.

## Method

### Participants

This study was part of a longitudinal pan-Canadian research project (the Pathways in Autism Project; http://www.asdpathways.ca) that examines the development of children on the autism spectrum. To date, data have been collected for 11 time points (Time 1 to Time 11) from five sites across Canada (Halifax, Montréal, Hamilton, Edmonton, and Vancouver). For the current study, we focused on data from 166 participants who completed the *Expression, Reception and Recall of Narrative Instrument* (ERRNI, Bishop 2004) at Time 8, when the children were between 10.5 and 11 years of age. Of those, 12 children were excluded as they did not complete the *Wechsler Intelligence Scale for Children-Fourth Edition* (WISC-IV; Wechsler 2003) at Time 6 (when they were between 8.5 and 9 years old), which resulted in a total of 154 participants.

All children received a diagnosis of an autism spectrum disorder as preschoolers, based on the Diagnostic and Statistical Manual of Mental Disorders, Fourth Edition, Text Revision (DSM-IV; APA, 2013) and through both the Autism Diagnostic Interview-Revised (ADI-R) and the Autism Diagnostic Observation Schedule (ADOS), administered by examiners who had reached research reliability on these measures. Additionally, diagnosis was confirmed through the ADOS at Time 8. Children were excluded from the Pathways in Autism project if they did not speak French or English, or if they were diagnosed with a genetic disorder or a neurological disorder that would prevent participation in testing. As shown in Table 1, at the time of assessment, children were 128 months (10.7 years) of age.

**Table 1:** Demographic information by language group. Table includes minimum, maximum and mean score, as well as standard deviation (SD)

Language Status		Min.	Max.	Mean	SD
Monolinguals	Age at assessment (months) at T8	121.71	137.06	128.59	2.67
	Highest level of education of the primary caregiver at T8	1	13	7.33	3.46
	WISC-IV verbal comprehension composite score at T6	47	124	85.30	17.07
	WISC-IV perceptual reasoning composite score at T6	56	126	92.50	18.62
	Proportion Exposure to language of testing at T8	0.96	1.00	1.00	0.01
Bilinguals	Age at assessment (months) at T8	125.72	134.76	128.94	2.06
	Highest level of education of the primary caregiver at T8	1	13	7.38	3.05
	WISC-IV verbal comprehension composite score at T6	47	134	88.62	19.76
	WISC-IV perceptual reasoning composite score at T6	56	134	97.87	20.55
	Proportion exposure to language of testing at T8	0.42	0.94	0.764	0.141

Monolinguals n = 80

Bilinguals n = 54

### Language status and language exposure

Children were identified as bilingual or monolingual based on caregivers’ answers on the *Family Background Information Questionnaire* (FBIQ) and the *School Services Questionnaire* (SSQ; Colli et al., 2009). The FBIQ is a caregiver questionnaire used to gather demographic information about the family, including the child’s language exposure. Caregivers were asked to identify whether the child received exposure to one or more languages at home, and to estimate the percentage of exposure that the child received to each language. The SSQ is a caregiver questionnaire used to gather information regarding school services. It includes information about the number of hours during which the child attended school each week, the language(s) in which the child was schooled, and an estimate of the amount of exposure that the child received to each language of schooling during class time. It should be noted that our “language exposure” variable is a best estimate of the amount of language that children hear and use. The FBIQ and the SSQ do not differentiate between input (what is heard) and output (what is said). However, Bedore et al., (2012) did not find a statistically significant difference in children’s input and output when language exposure information was gathered through parent questionnaires. Additionally, the use of parent questionnaires to measure language exposure is a common method in bilingualism literature (Bedore et al., 2012; Thordardottir, 2011, 2015, 2019; Unsworth, 2016; Beauchamp et al., 2020; Beauchamp & MacLeod, 2022).

Based on caregivers’ answers, current (i.e., at Time 8) language exposure proportion estimates were calculated. To do so, we calculated the number of hours of exposure that children received to each of their languages at home and at school separately on a weekly basis. Next, we multiplied those hours by 40 weeks for school exposure and by 52 weeks for home exposure. Finally, those totals were added for each language and a proportion was calculated by dividing the total number of hours in one language by the total number of language hours.

A language status variable (bilingual/monolingual) was created based on children’s language exposure and children were assigned to one of two groups. Following Beauchamp & MacLeod (2022) and Thordardottir (2011; 2019), children were included in the bilingually exposed group if parents reported that they currently received at least 5% exposure to a second language. While there is no accepted cut-off for the bilingual/monolingual differentiation (and this cut-off may vary depending on the child’s age and years of exposure to a second language), findings from Beauchamp (2020) suggest that 5% was the lowest amount of second-language exposure received by children who were identified by their parents as bilingual. Current exposure was chosen over lifetime exposure as it has been shown to be more strongly associated with children’s language abilities once they reach school age (Gonzalez Barrero & Nadig, 2018; Unsworth 2016). In cases for which caregivers did not indicate the proportion of exposure that the child received at home at T8, information from the nearest two timepoints (between T5 and T7) was averaged. Additionally, children were required to begin second language (L2) exposure by T6 in order to ensure that they had received at least 2 years of exposure to their second language at the time of testing.

Finally, for bilingual children for whom the proportion of exposure information was not available for school hours, only the proportions of exposure reported at home were used. This was deemed an appropriate option as school exposure represents only approximately 30% of a child’s time when we consider weekends, holidays, and summer breaks. In contrast, monolingually exposed children were defined as those children who had received less than 5% exposure to a second language (following Thordardottir 2019, and Beauchamp & MacLeod 2022 2020.An additional 20 children were excluded from the study because of missing information.

Based on these criteria (monolingual/bilingual), a total of 134 participants were included in the study: 54 bilingually exposed children, and 80 monolingually exposed children. For ease of reading, we will use *bilingual* to refer to bilingually exposed children and *monolingual* to refer to monolingually exposed children. This terminology reflects Grosjean’s (1993) definition of a bilingual as an individual who can use a second language, without stipulating a specific level of proficiency. Additionally, 11 children in the bilingual group received exposure to a third language. Although all children spoke either French or English, bilingual children also spoke a wide variety of other languages including (but not limited to) French, English, Mandarin, Arabic, Portuguese, Spanish, Urdu, Greek and Farsi. Information regarding children’s language exposure, verbal and non-verbal cognitive abilities, age of assessment, and other demographic information by language group can be found in Table 1. Notably, both groups include children with a wide range of cognitive and verbal abilities.

### Measures and Procedure

#### Narrative language

Narrative abilities were assessed using the ERRNI, a normed instrument that measures expressive and receptive narrative abilities in children. To assess macrostructure, two scores were calculated. The first score, *Storytelling-initial*, represents the child’s ability to tell a story that includes all the important events and characters in the story depicted in a wordless picture book. The second score, *Storytelling-recall*, represents the child’s ability to retell the same story, including as many events and characters as possible, and is completed without the picture support, approximately 15 min after the initial storytelling task. The ERRNI was completed either in French or in English, based on the child’s preference. Children’s stories were transcribed and scored following guidelines in the administration manual (see Volden et al., 2017, for a description of transcription and scoring methodology).

The ERRNI also provides measures of microstructure. For this study the following scores were calculated based on the transcriptions of children’s productions and following guidelines in the administration manual: (1) Storytelling-initial number of words, (2) Storytelling-initial number of utterances, (3) Storytelling-recall number of words, (4) Storytelling-recall number of utterances, and (5) total mean length of utterance-word (MLUw) standard score. The MLUw score is derived by dividing the total number of words produced by the number of utterances produced. It represents the complexity of children’s utterances (see Volden et al., 2017, for detailed description).

#### Pragmatic Language

Pragmatic language skills were assessed using the *Comprehensive Assessment of Spoken Language* (CASL; Carrow-Woolfolk 2001), a standardized measure that assesses children’s spoken language abilities. Specifically, we used the standard scores for three subtests: the *Nonliteral Language* subtest, the *Inferencing* subtest, and the *Pragmatic Judgement* subtest. During the Nonliteral Language subtest, participants are presented with a short scenario that contains nonliteral language and are asked to explain its meaning. The Inferencing subtest requires that participants listen to short scenarios from which information is missing. The participant must use his/her knowledge of the world to make inferences regarding the information that was omitted. The Pragmatic Judgement subtest requires that participants attend to a vignette about everyday situations and judge whether the characters in the story used appropriate language and made appropriate pragmatic judgments.

#### Social skills and intellectual ability

Social skills were assessed using the Vineland Adaptive Behavior Scales (VABS-II, Sparrow et al., 2005), Survey Interview, Socialization domain scores. The VABS is a caregiver interview frequently used both clinically and in research to examine the adaptive development of children across several domains. In line with Volden et al., (2017), the Socialization domain standard scores were used. This subscale examines children’s abilities to interact with others and to use social skills in a variety of situations. Intellectual ability was measured using the Weschler Intelligence Scales for Children - Fourth Edition (WISC-IV). As in Volden et al., (2017), we chose to use the *Perceptual Reasoning Index* (PRI) as a measure of non-verbal IQ (NVIQ). Children’s verbal IQ was assessed using the WISC-IV Verbal Comprehension Index.

At each site, children were tested in a quiet room at a research site or, less frequently, in the child’s school or home. Assessments were completed by a psychologist, psychometrist or trained research assistant. Parental consent was obtained at the beginning of each phase of the Pathways in Autism study and youth gave assent at the time of testing. Testing was completed in French or in English, although if francophone children could complete the narrative task in English, the ERRNI was completed in that language, regardless of whether it was the child’s dominant language.

### Analyses

Testing and scoring of narrative skills, pragmatic skills, social skills, and cognitive abilities were completed following the instructions in the relevant testing manuals. Table 2 provides scores by language group for the VABS and the CASL subtests.

**Table 2:** Standardised scores on the VABS, the ERRNI and the CASL by language group. Table includes minimum, maximum and mean scores, as well as standard deviation (SD)

	Monolinguals				Bilinguals			
	Min.	Max.	Mean	SD	Min.	Max.	Mean	SD
VABS Socialization SS	55	109	79.35	12.631	53	112	79.20	12.845
ERRNI Storytelling-initial: N words	60	411	157.61	58.595	68	256	142.50	46.080
ERRNI Storytelling-initial: N utterances	10	39	18.58	4.738	11	25	17.04	3.539
ERRNI Storytelling-recall: N words	0	276	118.10	59.574	4	267	119.45	60.462
ERRNI Storytelling- recall: N utterances	0	28	13.52	5.877	1	27	13.83	5.441
ERRNI Storytelling-initial SS	65	125	83.51	14.783	65	114	86.20	14.773
ERRNI Storytelling-recall SS	65	125	85.13	16.113	65	122	87.93	17.689
ERRNI MLUw SS	64.90	135.00	96.161	15.256	64.90	135.10	95.745	15.638
CASL Nonliteral Language SS	65	121	87.63	13.637	67	118	92.27	13.012
CASL T8 Pragmatic Judgement SS	40	115	76.59	18.240	40	121	79.98	19.176
CASL T8 Inference SS	52	108	83.82	13.314	52	115	83.64	17.470

Monolingual n = 80

Bilingual n = 54

### Analytic approach

For some statistical analyses, we used Bayesian statistics and Bayes Factors to compare group means. In contrast to classical null hypothesis significance testing, calculating the Bayes Factor allows “researchers to quantify how much more likely the data under one hypothesis in comparison to another and […] allowing researchers to non-directionally test this likelihood (i.e., evidence can be quantified in favour of the null hypothesis over the alternate, or vice versa”; Brydges & Gaeta 2019, p. 4524). In other words, Bayesian statistics give us a probability of likelihood that either the null hypothesis or the alternative hypothesis are confirmed. By contrast, classical significance testing only evaluates whether the null hypothesis can be rejected, and classical equivalence tests only evaluate whether the alternative hypothesis can be rejected. Bayesian statistical analyses are particularly relevant for comparing groups such as bilingual and monolingual children for whom the literature indicates both differences and similarities. In the following analyses, a Bayes Factor above 3.0 indicates that the null hypothesis is confirmed, while a Bayes Factor under 0.333 indicates that the alternative hypothesis is confirmed. Bayes Factors between 0.333 and 3.0 fall into the anecdotal range and confirm neither hypothesis (Brydges & Gaeta, 2019).

## Results

We examined whether children in the bilingual and monolingual groups differed on any demographic variables listed in Table 1. As Table 3 indicates, the language groups did not differ by age, verbal IQ, or NVIQ.

**Table 3:** T-tests comparing bilingual and monolingual group differences on demographic variables

	t	df	Sig
Highest level of education of the primary caregiver	− 0.082	127	0.935
Age at assessment (months)	− 0.807	132	0.421
VIQ composite score (T6)	-1.011	126	0.314
NVIQ composite score (T6)	-1.570	132	0.119
Proportion exposure to language of testing	14.710	132	0.000

Monolingual n = 80

Bilingual n = 54

Independent samples Bayesian t-tests were conducted to compare the scores of bilinguals and monolinguals on measures of macrostructure and microstructure. When examining macrostructure abilities, the analyses did not reveal a statistically significant group difference for either Storytelling-initial or Storytelling-recall (Storytelling initial: *t* = 1.032; *p* = 0.304; Bayes Factor_01_ = 4.417, Storytelling-recall *t* = 0.931; *p* = 0.353; Bayes Factor_01_= 4.781). Additionally, the respective Bayes Factors supported the null hypotheses.

When examining microstructure measures, there was a statistically significant group difference for Storytelling-initial number of utterances (*t*= -2.032; *p* = 0.044;), although the Bayes Factor (Bayes Factor_01_ = 1.052) fell in the anecdotal range suggesting that neither the null hypothesis nor the alternative hypothesis was supported. No other statistically significant difference was revealed (Storytelling-initial number of words: *t*= -1.591; *p* = 0.114; Bayes factor_01_ = 2.214; Storytelling-recall number of words *t* = 0.127; *p* = 0.899; Bayes factor_01_ = 7.213; Storytelling-recall number of utterances *t* = 0.307; *p* = 0.759; Bayes factor_01_ = 6.950; MLUw *t*= -0.422; *p* = 0.673; Bayes factor_01_ = 6,728) and the Bayes Factors supported the null hypothesis, with the exception of Storytelling-initial number of words, which fell in the anecdotal range. Additionally, when correcting for Type I errors for the two results in the anecdotal range (Storytelling-initial number of utterances and Storytelling-initial number of words) using Holms-Bonferroni correction, none of the group comparisons reached the significance threshold level (Storytelling-initial number of utterances *p* = 0.220; Storytelling-initial number of words *p =* 0.576).

Our previous analysis considered bilingualism/monolingualism as a binary variable; however, given the importance of language exposure for language abilities, we were interested in examining whether the amount of exposure that children received played a role in their performance. We completed a series of post hoc Pearson correlation analyses comparing the performances of children on various measures of macrostructure and microstructure in relation to the amount (proportion) of exposure that they received to the language of testing. Results for macrostructure indicate that scores for the Storytelling-recall and language exposure were close to the significance level (Storytelling-recall *r*= -0.173, *p* = 0.05), suggesting a trend toward an inverse relation between language exposure and macrostructure scores, and that scores for Storytelling-initial and exposure were non-significant (Storytelling-initial *r*= -0.140, *p* = 0.106;). When examining microstructure measures, results revealed no significant correlation between scores on microstructure measures and the amount of exposure to the language of testing (Storytelling-initial number of words: *r* = 0.001, *p* = 0.994; Storytelling-initial number of utterances: *r* = 0.047, *p* = 0.589; Storytelling-recall number of words *r*= -0.085; *p* = 0.330; Storytelling-recall number of utterances *r*= -0.102; *p* = 0.243). These results suggest that the amount of exposure that children in this group received to the language of testing did not significantly influence their microstructure scores.

We also examined whether bilingual and monolingual children performed differently on scores measuring social skills and pragmatic language. Again, we completed an independent-samples Bayesian t-test but this time with scores on the VABS and CASL as the dependent variables, and language group as the independent variable. None of the results was statistically significant (Nonliteral Language *t* = 1.801, *p* = 0.074 Bayes factor_01_ = 1,482; Pragmatic Judgement *t* = 0.975, *p* = 0.331, Bayes factor_01_ = 4.428; Inferential *t*= -0.052, *p* = 0.959; Bayes factor_01_ = 5.828; VABS Socialization Score *t*= -0.067, *p* = 0.946; Bayes factor_01_ = 7.140). Additionally, Bayes Factors supported the null hypothesis, indicating that the groups did not differ from one another, with the exception of the Nonliteral Language measure, which fell in the anecdotal range.

Next, since the previous analysis considered bilingualism/monolingualism as a binary variable, and given our interest in understanding whether language exposure played a role in social and pragmatic abilities, we completed a series of post hoc analyses to examine whether the amount of exposure to the language of testing that children received played a role in their social skills and pragmatic language abilities. To that end, Pearson’s correlations were completed to examine the association between the proportion of language exposure that children received to the language of testing and scores on the VABS-Socialization domain and on the CASL. Results show a significant negative correlation between language exposure and Nonliteral Language (*r*= -0.234, *p* = 0.012). There was also a trend toward a significant negative correlation between Pragmatic Language and exposure (*r*= -0.175, *p* = 0.54), but not for Inferencing (*r*= -0.059, *p* = 0.606) or for VABS Socialization score (*r* = 0.014, *p* = 0.811).

Finally, given the link between narrative abilities and social skills (Volden et al., 2017), we sought to examine the relation between macrostructure and microstructure on the one hand, and social skills and pragmatic language on the other hand. Two hierarchical regressions were completed with storytelling measures (i.e., Storytelling-initial and Storytelling-retell) as the dependent variables, scores on the VABS-Socialization domain and on the three measures of the CASL as the independent variables, and controlling for NVIQ and language abilities. Additionally, given the results of our post hoc analyses, we were also interested in examining the influence of language exposure. Since NVIQ and language abilities have previously been reported to influence narrative abilities (Volden et al., 2017), we entered scores from the PRI and MLUw in the first (Storytelling-initial) and second (Storytelling-retell) models, respectively. The proportion of language exposure that children received to the language of testing was entered last. Table 4 shows the results from the analyses using Storytelling-initial and Storytelling-recall as the dependent variables. When examining the Storytelling-initial analysis, once all of the variables were entered into the model, the only significant association was between scores on Storytelling-initial and MLUw. Social skills. Pragmatic language and language exposure were not significantly associated with Storytelling-initial scores. Interestingly, while there was an initial association between NVIQ and Storytelling-initial scores, it no longer reached significance when the CASL was entered into the model.

**Table 4:** Regressions with Storytelling-initial and Storytelling-recall and standard scores on MLUw, the VABS Socialization subtest and the CASL

Measures	Variables	Model 1			Model 2			Model 3			Model 4			Model 5		
		*B*	*SE B*	*β*	*B*	*SE B*	*β*	*B*	*SE B*	*β*	*B*	*SE B*	*β*	*B*	*SE B*	*β*
Storytelling-initial	Constant	59.444***	8.412		27.329*	10.394		24.543*	10.535		18.058	11.998		18.105	14.409	
	NVIQ	0.280***	0.083	0.372	0.240**	0.075	0.320	0.159	0.099	0.211	0.125	0.103	0.167	0.125	0.105	0.167
	MLUw				0.368***	0.083	0.441	0.321**	0.098	0.385	0.318**	0.097	0.382	0.318**	0.098	0.382
	Nonliteral Language							0.262	0.196	0.262	0.288	0.197	0.287	0.288	0.199	0.287
	Pragmatic Judgement							0.152	0.162	0.163	0.113	0.165	0.121	0.113	0.168	0.121
	Inferencing							− 0.250	0.146	− 0.274	− 0.239	0.146	− 0.262	− 0.239	0.148	− 0.262
	VABS										0.125	0.111	0.120	0.125	0.113	0.120
	Language exposure													− 0.049	8.207	− 0.001
	*R* ^*2*^	0.139			0.330			0.377			0.389			0.387		
	*F*	11.260***			17.015***			8.001***			6.904***			5.826***		
	Δ*R2*	0.139			0.192			0.047			0.012			0.000		
	Δ*F*	11.260***			19.753***			1.665			19,259			0.000		
Storytelling-recall	Constant	44.505***	9.782		11.447	12.483		3.472	12.439		1.648	14.256		8.564	17.086	
	NVIQ	0.448***	0.097	0.486	0.406***	0.089	0.441	0.209	0.116	0.227	0.199	0.122	0.216	0.189	0.124	0.205
	MLUw				0.380***	0.100	0.368	0.278*	0.116	0.289	0.277*	0.117	0.268	0.282*	0.117	0.273
	Nonliteral Language							0.584*	0.231	0.474	0.591*	0.234	0.479	0.585*	0.235	0.474
	Pragmatic Judgement							− 0.167	0.192	− 0.146	− 0.177	0.197	− 0.156	− 0.197	0.200	− 0.173
	Inferencing							− 0.023	0.173	− 0.020	− 0.020	0.174	− 0.018	− 0.005	0.176	− 0.005
	VABS										0.035	0.132	0.028	0.048	0.134	0.037
	Language exposure													-7.197	9.723	− 0.072
	*R* ^*2*^	0.236			0.370			0.436			0.436			0.441		
	*F*	21.332***			19.957***			10.030***			8.251***			7.101***		
	Δ*R2*				0.134			0.066			0.001			0.001		
	Δ*F*				14.431***			2.520			0.072			0.548		

Note : p = 0.001***, p = 0.01**, *p =* 0.05*

Storytelling-initial n = 81 df Model 1 (1, 80); Model 2 (2, 80); Model 3 (5, 80); Model 4 (6, 80); Model 5 (7, 80)

Storytelling-recall n = 79 df Model 1 (1, 78); Model 2 (2, 78); Model 3 (5, 78); Model 4 (6, 78); Model 5 (7, 78)

As Table 4 shows, when examining the Storytelling-recall analysis, there was a significant association between MLUw and the Storytelling-recall scores. Additionally, there was a significant association between Storytelling-recall and Nonliteral Language scores on the CASL. Again, neither social skills nor language exposure was significantly associated with Storytelling-recall scores. Moreover, while there was initially an association between NVIQ and the Storytelling-recall scores, the association non-significant once the CASL was entered into the model.

Finally, we examined the relation between microstructure and social skills and pragmatic language. Again, a hierarchical regression was completed using the *enter* function with MLUw standard scores as the dependent variable (following Volden et al., 2017), scores on the VABS-Socialization domain, scores on the three measures of the CASL, and exposure to the language of testing as independent variables, and controlling for NVIQ. Results (Table 5) indicate that the model became statistically significant when the CASL measures were entered together. However, individual predictors did not contribute to the model in a statistically significant way. Upon examining collinearity, although the tolerance level did not reach the 0.1 threshold, it was nevertheless considered to be somewhat weak (Nonliteral language = 0.252; Pragmatic Judgement = 0.323; Inference = 0.375). We therefore re-ran the same regression model including solely Pragmatic Judgement as the pragmatic language predictor. As Table 6 indicates, this new set of regressions revealed the same general pattern but included a more accurate calculation of individual predictors, indicating that the Pragmatic Judgement measure was a significant component of the model and significantly correlated with MLUw.

**Table 5:** Regressions with MLUw and standard scores on the VABS Socialization subtest and the CASL

Measure	Variables	Model 1			Model 2			Model 3			Model 4		
		*B*	*SE B*	*β*	*B*	*SE B*	*β*	*B*	*SE B*	*β*	*B*	*SE B*	*β*
MLUw	Constant	87.385***	10.798		50.972***	11.629		49.157***	13.900		43.821*	17.335	
	NVIQ	0.108	0.107	0.119	− 0.248*	0.120	− 0.275*	− 0.257*	0.126	− 0.285	− 0.248	0.128	− 0.275
	Nonliteral Language				0.288	0.243	0.239	0.295	0.246	0.245	0.298	0.248	0.247
	Pragmatic Judgement				0.315	0.199	0.282	0.304	0.205	0.272	0.318	0.208	0.285
	Inferencing				0.231	0.181	0.212	0.234	0.183	0.214	0.222	0.185	0.203
	VABS							0.034	0.140	0.027	0.025	0.142	0.020
	Language exposure										5.374	10.325	0.055
	*R* ^*2*^	0.014			0.312			0.312			0.315		
	*F*	1.013			7.588***			5.997***			4.988***		
	Δ*R2*				0.298			0.001			0.003		
	Δ*F*				9.655***			0.059			0.271		

Note : p = 0.001***, p = 0.01**, *p =* 0.05*

df Model 1 (1, 71); Model 2 (2, 71); Model 3 (5, 71); Model 4 (5, 71)

n = 72

**Table 6:** Regressions with MLUw and standard scores on the VABS Socialization subtest and the Pragmatic Judgement subtest

Measure	Variables	Model 1 df (1,112)			Model 2 df (2,112)			Model 3 df (3,112)			Model 4 df (4,112)		
		*B*	*SE B*	*β*	*B*	*SE B*	*β*	*B*	*SE B*	*β*	*B*	*SE B*	*β*
MLUw	Constant	79.537***	7,227		69.481***	7.054		59.822	9.355		53.297***	13.176	
	NVIQ	0.173*	0.074	0.217	− 0.057	0.086	− 0.072	− 0.089	0.087	− 0.111	− 0.080	0.089	− 0.101
	Pragmatic Judgement				0.410***	0.092	0.479	0.391***	0.092	0.457	0.396***	0.093	0.463
	VABS							0.178	0.114	0.144	0.170	0.115	0.137
	Language exposure										6.574	9.327	0.062
	*R* ^*2*^	0.047			0.193			0.210			0.214		
	*F*	5.504*			13.134***			9.680***			7.350***		
	Δ*R2*				0.146			0.018			0.004		
	Δ*F*				19.830***			2.430			0.497		

Note : p = 0.001***, p = 0.01**, *p =* 0.05*

df Model 1 (1, 112); Model 2 (2, 112); Model 3 (3, 112); Model 4 (4, 112)

n = 113

## Discussion

Previous studies have shown a link between social skills and narrative abilities in children on the autism spectrum (Volden et al., 2017), and between pragmatic abilities and narrative skills (Ketelaars et al., 2016). Other studies have also found a relation between bilingualism and social skills in younger children on the autism spectrum (Hambly & Fombonne, 2012). However, it was unclear how bilingualism, social skills, and pragmatic language together influenced the performance of school-aged children on the autism spectrum on narrative tasks, and whether these children maintained a bilingual advantage (i.e., better skills linked to being bilingual) in the social domain as they reached school-age. Our goal for this study was therefore to examine the narrative abilities, social skills, and pragmatic language aptitudes of school-aged bilingual and monolingual children on the autism spectrum, and the possible relations among these variables.

Our findings indicate that bilingual children in this sample had similar performances on tasks measuring macrostructure compared to those of their monolingual peers (as indicated not only by the *p* values but also by the Bayes Factors). However, contrary to our predictions, we did not find a bilingual advantage for macrostructure abilities when compared to those of their monolingual peers. Our findings for macrostructure and microstructure are similar to those in Huang et al. (2018), the only other study to our knowledge to examine the influence of bilingualism on narrative skills in children on the autism spectrum. These findings suggest that bilingual children on the autism spectrum in this study reached similar levels of proficiency to those of their monolingual peers on measures of microstructure and macrostructure.

Additionally, language exposure did not significantly influence these bilingual children’s performances on either macrostructure or on microstructure measures. Although this finding is not surprising for macrostructure, it is for microstructure, and may be driven by variations in language exposure profiles. As Table 1 shows, in the current sample, the amount of exposure that bilingual children received to the language of testing ranged from 42 to 94%. Therefore, in line with previous findings in ND bilingual children (Thordardottir, 2011, 2015, 2019), our findings suggest that school-aged children on the autism spectrum who are either *balanced* bilinguals (i.e., receiving somewhat similar amounts of exposure to their two languages) or *high-low* bilinguals (i.e., high exposure to one language and low to the other) assessed in the language in which they received the most exposure, can reach levels of proficiency similar to those of their monolingual peers, when examining structural language and narrative skills.

When examining the bilingual advantage in the domains of social skills and pragmatic language, our findings differed from previous studies with younger children on the autism spectrum (Hambly & Fombonne, 2012; Valicenti-McDermott et al., 2015), as we did not find a bilingual advantage for social abilities. We did, however, find such an advantage for pragmatic language skills. That is, children’s performances on the Nonliteral Language subtest of the CASL (and less significantly on the Pragmatic Judgement subtest) were inversely related to the amount of exposure that children received. Given our bilingual distribution (42–94% exposure to the language of testing), this finding indicates that balanced bilinguals had stronger performances on the test measuring nonliteral language abilities. Our findings highlight that the bilingual advantage might be modulated by the language exposure patterns of the children in this study. Previous studies have shown that being a balanced bilingual can lead to improved executive functioning when compared to non-balanced (i.e., *high-low*) bilinguals or to monolinguals (Vega & Fernandez, 2011). In bilinguals, both language systems are constantly activated; therefore, bilinguals must inhibit interference from one language system when they are using the other (see Bialystok 2015, for discussion). Balanced bilinguals are more likely to switch frequently from one language system to another compared to children with *high-low* language exposure patterns and may therefore be required to use executive functioning more often. It is possible that balanced bilinguals’ frequent use of executive function supports their ability to inhibit the literal meaning of an utterance and to think flexibly beyond an utterance’s literal meaning, thus permitting bilingual children on the autism spectrum to understand nonliteral language more easily than their monolingual peers. Further studies will be required to confirm this pattern.

We also examined the link between narrative abilities and social skills, as well as pragmatic language for our entire sample (i.e., bilingual and monolingual children). While we did not find a relation between storytelling abilities and social skills (as reported in Volden, 2017), we did find a relation between pragmatic language abilities and children’s proficiency on some aspects of narrative skills. Specifically, pragmatic language skills were associated with language complexity, namely MLUw scores. This relation is likely explained by the need for strong language abilities required to complete pragmatic language tasks, rather than the influence of pragmatic language on language complexity scores. We also found a relation between nonliteral language skills and the story recall task, but not the storytelling task. It is unclear why only story recall was influenced by abilities in nonliteral language. It is possible that this reflects the need for working memory for both the story recall and nonliteral language tasks (Gabid, 2008; Qualls & Harris, 2003).

Our findings are particularly important in that our sample included children with lower language and cognitive abilities, as well as children with higher language and cognitive abilities. Consequently, our findings represent the language, social, and pragmatic abilities of school-aged bilingual children from a wide range of children on the autism spectrum, which is an additional novel contribution to the field of narrative and bilingualism in children on the autism spectrum. However, further studies replicating these findings are required in order for these findings to be generalizable.

## Clinical implications

From a clinical perspective, our findings indicate that children on the autism spectrum can benefit from a bilingual advantage in pragmatic abilities provided that they receive more balanced exposure to both of their languages. Our findings also indicate that bilingual children on the autism spectrum have the potential to reach monolingual levels of proficiency during natural language tasks such as the production of narratives. This is particularly true for balanced bilinguals and high-low bilinguals tested in their stronger language. Thus, clinicians can support and encourage dual-language exposure in children on the autism spectrum. However, results from this study also indicate that bilingualism is not a dichotomous variable. Clinicians are therefore encouraged to keep this in mind when assessing the language abilities of all bilingual children.

## Limitations and future directions

This study has several limitations. First, our aim was to examine microstructure and macrostructure, in a manner similar to how narratives are often assessed clinically. Consequently, we used the scores from the ERRNI rather than completing an in-depth transcript analysis of macrostructure and microstructures. However, an in-depth examination of children’s productions could shed light on specific areas of differences between bilinguals and monolinguals and future studies should consider this type of analysis.

Second, social skills were measured using a parent questionnaire rather than a direct assessment measure, which could have yielded more fine-grained results. For example, we were unable to specifically examine the link between communication skills such as proficiency in conversational skills and narrative skills. Thus, future studies may want to examine the link between narrative abilities and specific social communication skills. A third limitation is that this study only included children who were able to complete the ERRNI. While the children in this sample had a wide range of cognitive abilities, this sample is not representative of the entire autism spectrum. A fourth limitation is the way in which language exposure was measured. Calculations of the amount of exposure that children received were based on the overall amount of exposure that parents reported for their children at home and at school. Consequently, this calculation did not consider exposure outside of those two environments, nor the language in which children received intervention. Future studies should ensure to include a more robust measure of language exposure. Additionally, we included in our sample children who received as little as 5% exposure to another language. This methodological choice may have had an influence on the findings from our binary (bilingual/monolingual) analyses.

Moreover, data were not gathered concerning parents’ language proficiency (in either of their languages) and intellectual abilities. Future studies may consider reporting these measures as they may influence children’s language abilities.

A final limitation is that our study did not include either ND children or children with a DLD or intellectual disability. It would also be interesting to compare the scores of bilingual and monolingual children on the autism spectrum to those of children with other diagnoses. Such analyses would shed light on differences across these different diagnostic groups.

## Conclusions

This study is the first large-scale study to examine the narrative, social, and pragmatic language skills of bilingual children on the autism spectrum and to examine the link between narrative abilities and both social skills and pragmatic language aptitudes. Our findings show that bilingualism was not a risk factor for the children in our study and that it may in fact confer advantages in the domain of pragmatic language. However, these advantages are not present in all bilinguals; rather, they were modulated by the amount of dual-language exposure that a child received. Thus, the more evenly balanced exposure to their languages, the more likely children seem to experience a bilingual advantage. These findings thus serve to improve our understanding of narrative and pragmatic abilities in bilingual children on the autism spectrum, to inform clinicians who work with bilingual children, and to support caregivers who want or need to raise their children in a bilingual context. From a theoretical perspective, these findings improve our understanding of the bilingual advantage and may shed light on the different factors that influence this advantage.
